# Differences in Health Professionals’ Engagement With Electronic Health Records Based on Inpatient Race and Ethnicity

**DOI:** 10.1001/jamanetworkopen.2023.36383

**Published:** 2023-10-09

**Authors:** Chao Yan, Xinmeng Zhang, Yuyang Yang, Kaidi Kang, Martin C. Were, Peter Embí, Mayur B. Patel, Bradley A. Malin, Abel N. Kho, You Chen

**Affiliations:** 1 Department of Biomedical Informatics, Vanderbilt University Medical Center, Nashville, Tennessee; 2 Department of Computer Science, Vanderbilt University, Nashville, Tennessee; 3 Feinberg School of Medicine, Northwestern University, Chicago, Illinois; 4 Department of Biostatistics, Vanderbilt University Medical Center, Nashville, Tennessee; 5 Critical Illness, Brain Dysfunction, and Survivorship (CIBS) Center, Vanderbilt University Medical Center, Nashville, Tennessee; 6 Geriatric Research and Education Clinical Center, Veterans Affairs, Tennessee Valley Healthcare System, Nashville; 7 Division of Acute Care Surgery, Department of Surgery, Vanderbilt University Medical Center, Nashville, Tennessee,; 8 Department of Hearing and Speech Sciences, Vanderbilt University Medical Center, Nashville, Tennessee; 9 Institute for Public Health and Medicine, Northwestern University, Chicago, Illinois; 10 Department of Medicine-General Internal Medicine, Northwestern University, Chicago, Illinois

## Abstract

**Importance:**

US health professionals devote a large amount of effort to engaging with patients’ electronic health records (EHRs) to deliver care. It is unknown whether patients with different racial and ethnic backgrounds receive equal EHR engagement.

**Objective:**

To investigate whether there are differences in the level of health professionals’ EHR engagement for hospitalized patients according to race or ethnicity during inpatient care.

**Design, Setting, and Participants:**

This cross-sectional study analyzed EHR access log data from 2 major medical institutions, Vanderbilt University Medical Center (VUMC) and Northwestern Medicine (NW Medicine), over a 3-year period from January 1, 2018, to December 31, 2020. The study included all adult patients (aged ≥18 years) who were discharged alive after hospitalization for at least 24 hours. The data were analyzed between August 15, 2022, and March 15, 2023.

**Exposures:**

The actions of health professionals in each patient’s EHR were based on EHR access log data. Covariates included patients’ demographic information, socioeconomic characteristics, and comorbidities.

**Main Outcomes and Measures:**

The primary outcome was the quantity of EHR engagement, as defined by the average number of EHR actions performed by health professionals within a patient’s EHR per hour during the patient’s hospital stay. Proportional odds logistic regression was applied based on outcome quartiles.

**Results:**

A total of 243 416 adult patients were included from VUMC (mean [SD] age, 51.7 [19.2] years; 54.9% female and 45.1% male; 14.8% Black, 4.9% Hispanic, 77.7% White, and 2.6% other races and ethnicities) and NW Medicine (mean [SD] age, 52.8 [20.6] years; 65.2% female and 34.8% male; 11.7% Black, 12.1% Hispanic, 69.2% White, and 7.0% other races and ethnicities). When combining Black, Hispanic, or other race and ethnicity patients into 1 group, these patients were significantly less likely to receive a higher amount of EHR engagement compared with White patients (adjusted odds ratios, 0.86 [95% CI, 0.83-0.88; *P* < .001] for VUMC and 0.90 [95% CI, 0.88-0.92; *P* < .001] for NW Medicine). However, a reduction in this difference was observed from 2018 to 2020.

**Conclusions and Relevance:**

In this cross-sectional study of inpatient EHR engagement, the findings highlight differences in how health professionals distribute their efforts to patients’ EHRs, as well as a method to measure these differences. Further investigations are needed to determine whether and how EHR engagement differences are correlated with health care outcomes.

## Key Points

**Question:**

Are there differences in health professionals’ engagement with hospitalized patients’ electronic health records (EHRs) with respect to race and ethnicity?

**Findings:**

In this cross-sectional study of 243 216 adult patients hospitalized at 2 major US academic medical institutions between 2018 and 2020, the EHRs of minoritized racial and ethnic populations were more likely to receive less health professional engagement compared with those of White patients.

**Meaning:**

The findings highlight unequal EHR engagement by race and ethnicity in the inpatient setting, and the analytic methods introduced in this study can be used to assess institutional EHR usage patterns across patient subpopulations.

## Teaser

This cross-sectional study investigates differences in the level of health professionals’ engagement with electronic health records according to inpatient race and ethnicity.

## Introduction

Health care disparities continue to persist in the US medical system.^1,2^ Marginalized racial and ethnic groups,^3,4^ individuals with low income and limited access to health care and insurance,^5^ and others who are continuously subject to discrimination^6,7^ bear a disproportionate burden in fulfilling their needs of care.^1,8^ The effect of these disparities is compounded by the inherently higher risks of poor health outcomes for these groups.^9^

Recent studies have shown that clinicians in the US spend a significant amount of time (up to 56% of their active duty hours) engaging with patients’ electronic health records (EHRs) to deliver care.^10,11,12^ The quantity of EHR engagement reflects the amount of information documented and accessed by health professionals regarding the treatment of their patients. Growing evidence suggests that a higher level of EHR effort is associated with better outcomes for patients.^13,14,15,16^ However, it is unclear whether there are differences in the level of EHR engagement based on different patient races and ethnicities.

To investigate this issue, we reviewed the access log data documented in EHR systems, which objectively record the time-stamped behavior of health professionals with respect to their EHR use throughout a patient’s care.^17,18,19,20,21,22,23,24^ Our study focuses on approximately 243 000 adults who experienced an inpatient stay at 1 of 2 large, geographically distinct US academic medical institutions between 2018 and 2020 to characterize the differences in EHR engagement with respect to race and ethnicity. We hypothesized that there are differences in the level of EHR engagement by health professionals for hospitalized patients of different races and ethnicities.

## Methods

This retrospective cross-sectional study was approved by the institutional review boards (IRBs) at Vanderbilt University Medical Center (VUMC) and Northwestern Medicine (NW Medicine). A full waiver of written informed consent from patients was granted by both IRBs because this study is retrospective with minimal risk to patients, and the waiver does not compromise the patients’ rights or welfare. We conducted the research independently at the 2 institutions to verify the replicability of any findings that may emerge. This study follows the Strengthening the Reporting of Observational Studies in Epidemiology (STROBE) reporting guideline.

### Study Cohorts and Data

We conducted this study based on the inpatient hospital stays of adults (aged ≥18 years) admitted to 2 major US academic medical institutions between January 1, 2018, and December 31, 2020: VUMC, a 1162-bed tertiary hospital in Nashville, Tennessee, and NW Medicine, a 2554-bed tertiary medical center with 11 sites distributed throughout Chicago, Illinois. Both institutions rely on the same EHR vendor system (ie, Epic Systems).

We excluded inpatient stays that ended in death or lasted less than 24 hours. For patients who experienced multiple admissions, we retained the first inpatient stay. We collected patient demographic and socioeconomic information as documented in the EHR, as well as the diagnoses associated with the inpatient stay. Demographic and socioeconomic factors were characterized by age, sex (female or male), self-reported race and ethnicity (Black, Hispanic, White, or other races and ethnicities [American Indian or Alaska Native, Asian, Asian Indian, Chinese, Filipino, Guamanian or Chamorro, Japanese, Korean, Native Hawaiian, Other Asian, Other Pacific Islander, Samoan, Vietnamese, and none of the above as derived from the EHR databases]), insurance type (public, private, or self-pay), and deprivation index (DI)^25^ based on a patient’s geographic area of residence. In this study, individuals who identified themselves as Hispanic in ethnicity were categorized as Hispanic, regardless of race. The “other race and ethnicity” category was used to denote the set of racial and ethnic groups with an insufficient number of patients to enable meaningful statistical analysis. The DI was incorporated as a covariate to control for socioeconomic factors so that we could quantify whether there were differences in EHR engagement among different racial and ethnic subpopulations with the same socioeconomic status. The DI applied in this study corresponds to a measure derived from the American Community Survey to represent the degree of community deprivation. This index was computed based on multiple census-level factors, including the poverty ratio, median household income, education level, insurance coverage, public assistance income coverage, and ratio of house vacancy.^25^ The DI ranges from 0 (least deprived) to 1 (most deprived). We defined a patient’s clinical status during their inpatient stay by the 17 condition categories of the Charlson Comorbidity Index,^26,27^ which characterizes health resource utilization.

The EHR access logs are mandated by the Health Insurance Portability and Accountability Act of 1996. They document the granular interactions of credentialed users with the EHR interface.^21^ A new access record is created each time a health professional views, modifies, or triggers a function of the EHR system within a patient’s EHR. This record includes a time stamp, user ID, patient medical record number, interaction type description, and other pertinent information. There were 1144 and 1228 distinct types of user-EHR interactions captured at VUMC and NW Medicine, respectively. Given the admission and discharge time of an inpatient stay, we collected the corresponding access log data from the enterprise Epic EHR database. The details of the inclusion and exclusion criteria are provided in eFigure 1 in Supplement 1.

### Study Design and Measures

We measured the level of inpatient EHR engagement by calculating the average number of user-EHR interactions performed with the patient’s EHR per hour throughout the duration of the inpatient stay. We derived this measure by dividing the total number of user-EHR interactions recorded in the EHR access log data by the corresponding length of stay (in hours). The EHR engagement metric quantifies the average number of EHR actions undertaken by health professionals each hour throughout a patient’s inpatient stay. This metric essentially measures the hourly EHR workload or attention devoted to a patient. It can also be interpreted as the frequency with which health professionals document and access a patient’s information from the EHR during their care. The categorical breakdowns of user-EHR interactions were also considered as additional outcome variables. Specifically, each user-EHR interaction belongs to 1 of the following 4 categories: view (eg, the problem list is viewed), modify (eg, pend a note), export (eg, operating room report printed), and system (eg, barcode scanned).^24^ System represents the remaining interactions that trigger the usage of a certain function of the EHR system. We then restricted our analysis to the categories of these user-EHR interactions and derived the corresponding outcome variables.

We first investigated potential differences in health professionals’ EHR engagement by racial and ethnic patient subpopulations over the 3-year period. We then replicated the analysis by calendar year to determine how engagement changed over time.

### Statistical Analysis

The data analysis was performed between August 15, 2022, and March 15, 2023. To investigate the differences in EHR engagement between racial and ethnic patient subpopulations, we first categorized the continuous outcome variable into 4 ordinal categories based on the 4 quartiles. These quartiles were specific to the investigation time window and the corresponding cohort. We then performed a proportional odds logistic regression analysis for each cohort, with patients’ demographic information (ie, age, sex), socioeconomic characteristics (ie, insurance type, DI), and comorbidities (ie, Charlson Comorbidity Index categories) adjusted, as represented in the conceptual framework (eFigure 2 in Supplement 1). We selected proportional odds logistic regression over linear regression because the dependent variable was bounded by 0, and there was nonconstant variance in the residuals of the linear regression. The reference values were White (for race and ethnicity), female (for sex), DI lower than 0.40 at VUMC and lower than 0.29 at NW Medicine (for DI), and public insurance (for insurance type). The thresholds for DI were the median values of DI distributions. Although length of stay was factorized into EHR engagement, it might still have a mediation effect between race and ethnicity and EHR engagement. Thus, we performed a sensitivity analysis by adding length of stay as an extra covariate to examine the robustness of the primary analysis results.

We also conducted unadjusted analyses to help with interpreting the results. Proportional odds assumptions were checked and confirmed through the graphical method due to the large cohort sizes.^28^ An adjusted odds ratio (AOR) of a factor greater than 1.0 suggests that this factor is associated with a higher odds of having more EHR engagement (ie, a higher category of the outcome variable in a general sense) compared with the reference group after adjusting for other factors. To characterize the difference changes over time, we used permutation tests to compare the AORs of non-White (ie, Black, Hispanic, other race and ethnicity) vs White patients for having a higher EHR engagement between calendar years. Specifically, the data points from any 2 years were shuffled and separated into 2 samples of the original sizes. The AORs of race and ethnicity were reestimated in each part, and their difference was calculated. The process was repeated 1000 times, and the 2-sided *P* value was calculated based on the distribution of the 1000 AOR estimate differences. A significance threshold of *P* < .05 was applied for all statistical tests. We performed all statistical analyses using R, version 4.1.2 software (R Foundation for Statistical Computing) and produced figures using Python, version 3.8 software (Python Software Foundation).

## Results

A total of 80 946 and 162 470 adult inpatient stays between 2018 and 2020 were included from VUMC and NW Medicine, respectively (eFigure 1 in Supplement 1). The VUMC cohort consisted of 54.9% female and 45.1% male patients; 14.8% were Black, 4.9% were Hispanic, 77.7% were White, and 2.6% patients were other races and ethnicities; the mean (SD) age was 51.7 (19.2) years (Table 1). The NW Medicine cohort consisted of 65.2% female and 34.8% male patients; 11.7% were Black, 12.1% were Hispanic, 69.2% were White, and 7.0% patients were other races and ethnicities. The mean (SD) age was 52.8 (20.6) years.

**Table 1.** Characteristics of Study Cohorts

Characteristic	No. (%) or mean (SD)							
	Vanderbilt University Medical Center				Northwestern Medicine			
	2018-2020 (n = 80 946)^a^	2018 (n = 28 858)	2019 (n = 30 482)	2020 (n = 31 740)	2018-2020 (n = 162 470)^a^	2018 (n = 54 993)	2019 (n = 62 239)	2020 (n = 67 875)
Race and ethnicity								
Black	11 975 (14.8)	4426 (15.3)	4705 (15.4)	4953 (15.6)	19 067 (11.7)	6619 (12.0)	7780 (12.5)	8049 (11.9)
Hispanic	3973 (4.9)	1244 (4.3)	1462 (4.8)	1658 (5.2)	19 638 (12.1)	6037 (11.0)	7210 (11.6)	8661 (12.8)
White	62 911 (77.7)	22 491 (77.9)	23 534 (77.2)	24 306 (76.6)	112 442 (69.2)	38 646 (70.3)	42 999 (69.1)	46 739 (68.9)
Other^b^	2087 (2.6)	697 (2.4)	781 (2.6)	823 (2.6)	11 323 (7.0)	3691 (6.7)	4250 (6.8)	4426 (6.5)
Sex								
Male	36 474 (45.1)	13 123 (45.5)	13 808 (45.3)	14 376 (45.3)	56 584 (34.8)	18 978 (34.5)	21 981 (35.3)	24 708 (36.4)
Female	44 472 (54.9)	15 735 (54.5)	16 674 (54.7)	17 364 (54.7)	105 886 (65.2)	36 015 (65.5)	40 258 (64.7)	43 167 (63.6)
Insurance type								
Private	30 023 (37.1)	10 481 (36.3)	11 032 (36.2)	11 379 (35.9)	80 007 (49.2)	26 619 (48.4)	29 526 (47.4)	31 124 (45.9)
Public	45 957 (56.8)	16 788 (58.2)	17 604 (57.8)	18 485 (58.2)	78 857 (48.5)	27 138 (49.3)	31 296 (50.3)	35 469 (52.3)
Self-pay	4966 (6.1)	1589 (5.5)	1846 (6.0)	1876 (5.9)	3606 (2.2)	1236 (2.2)	1417 (2.3)	1282 (1.9)
Age at admission, y	51.7 (19.2)	51.7 (19.4)	52.0 (19.1)	51.7 (19.2)	52.8 (20.6)	53.3 (20.7)	53.7 (20.6)	54.1 (20.7)
Length of stay, d	5.1 (6.6)	5.0 (6.0)	5.1 (6.3)	5.1 (6.3)	4.8 (5.6)	4.6 (5.2)	4.7 (5.2)	5.1 (6.1)
Charlson Comorbidity Index score	2.1 (2.7)	2.1 (2.7)	2.2 (2.7)	2.2 (2.7)	1.7 (2.4)	1.7 (2.5)	1.8 (2.5)	1.9 (2.6)
Deprivation index	0.39 (0.08)	0.39 (0.08)	0.39 (0.08)	0.39 (0.08)	0.31 (0.10)	0.31 (0.10)	0.31 (0.10)	0.31 (0.10)
EHR activity during inpatient stay								
Total No. of user-EHR interactions	15 597 (22 319)	16 799 (22 683)	13 926 (19 784)	15 240 (21 032)	14 609 (21 385)	16 932 (21 833)	13 379 (18 570)	14 027 (21 573)
No. of user-EHR interactions per hour	111.0 (36.8)	120.4 (34.6)	101.2 (34.7)	110.6 (38.4)	128.5 (51.0)	155.8 (56.3)	119.4 (47.4)	113.6 (36.6)
No. of modify actions per hour	6.9 (2.3)	6.9 (2.2)	6.9 (2.3)	6.9 (2.4)	9.0 (3.4)	8.9 (3.3)	9.1 (3.4)	9.0 (3.3)
No. of view actions per hour	76.7 (28.7)	88.3 (29.1)	71.0 (27.5)	69.8 (25.6)	108.3 (46.0)	132.0 (51.4)	100.0 (42.7)	95.9 (33.1)
No. of export actions per hour	20.3 (9.9)	18.5 (3.0)	17.4 (8.0)	25.5 (13.7)	7.1 (1.6)	6.7 (1.7)	7.3 (1.7)	7.4 (1.7)
No. of system actions per hour	7.1 (5.9)	6.7 (4.3)	5.9 (4.8)	8.4 (7.6)	4.2 (3.8)	8.3 (2.7)	3.1 (3.2)	1.4 (0.6)
Quartiles of No. of user-EHR interactions per hour								
1	87.8	99.6	79.4	87.3	92.49	115.59	86.41	89.27
2	110.7	120.1	99.4	110.4	118.18	149.18	109.37	108.27
3	134.1	141.0	122.3	135.0	155.36	188.53	143.01	132.82

Abbreviation: EHR, electronic health record.

^a^ The fact that the size of the 3-year cohort was slightly smaller than the sum of the sizes of the single-year cohorts was because a small fraction of patients existed in more than 1 single-year cohort.

^b^ Other race and ethnicity includes American Indian or Alaska Native, Asian, Asian Indian, Chinese, Filipino, Guamanian or Chamorro, Japanese, Korean, Native Hawaiian, Other Asian, Other Pacific Islander, Samoan, Vietnamese, and none of the above. These categories were derived from EHR databases.

**Figure 1.**
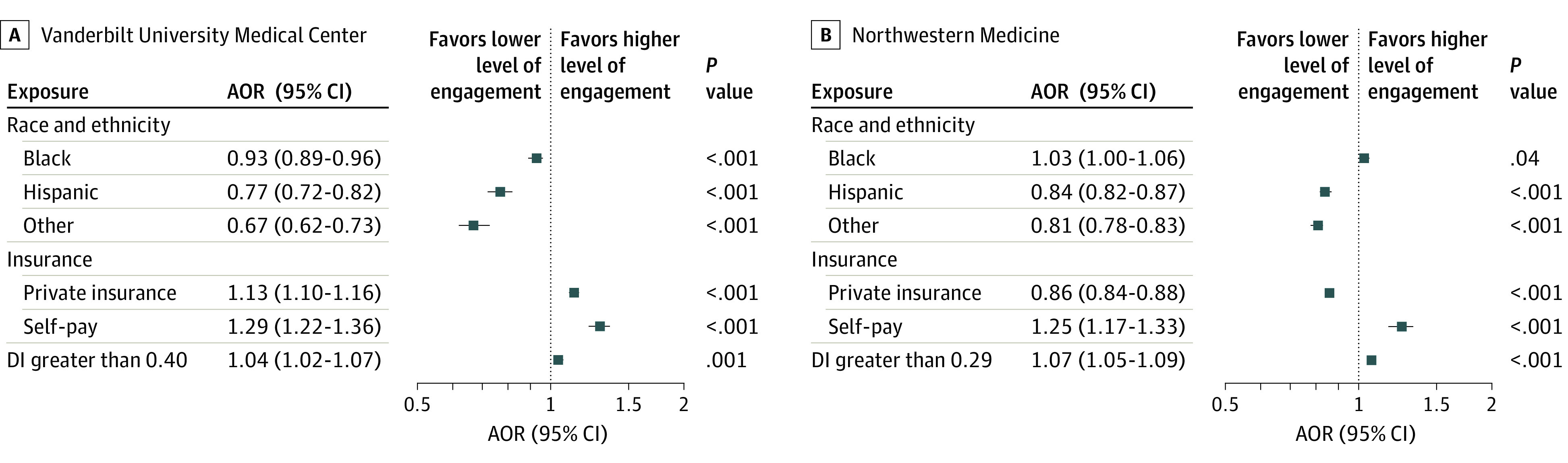
Adjusted Odds Ratios (AORs) of Patients’ Race and Ethnicity and Socioeconomic Factors for Electronic Health Record Engagement, 2018-2020 White race, public insurance, and deprivation index (DI) less than 0.40 for Vanderbilt University Medical Center and less than 0.29 for Northwestern Medicine are the reference groups for race and ethnicity, insurance type, and DI, respectively. Comorbidity categories were defined per Charlson Comorbidity Index; age and sex were adjusted.

Differences in EHR engagement between racial and ethnic subpopulations were observed in both adjusted and unadjusted 3-year analyses at the 2 institutions (Figure 1; eFigure 3 in Supplement 1). At VUMC, Black race (AOR, 0.93; 95% CI, 0.89-0.96; *P* < .001), Hispanic (AOR, 0.77; 95% CI, 0.72-0.82; *P* < .001), and other races and ethnicities (AOR, 0.67; 95% CI, 0.62-0.73; *P* < .001) were associated with a lower likelihood of a greater amount of EHR engagement compared with White. At NW Medicine, Hispanic ethnicity (AOR, 0.84; 95% CI, 0.82-0.87; *P* < .001) and other races and ethnicities (AOR, 0.81; 95% CI, 0.78-0.83; *P* < .001) were also associated with a lower likelihood of a greater amount of EHR engagement compared with White race, whereas the difference between Black (AOR, 1.03; 95% CI, 1.00-1.06; *P* = .04) and White was much smaller. Compared with public insurance, self-pay at both institutions was associated with a higher likelihood of a greater amount of EHR engagement, whereas private insurance showed contrary associations (ie, a higher likelihood of a greater amount of EHR engagement at VUMC and a lower likelihood at NW Medicine). A higher DI score (ie, a more deprived living environment) was also associated with a higher likelihood of a greater amount of EHR engagement at both institutions. A sensitivity analysis indicated that the aforementioned associations remained evident after further adjusting for the patient’s length of stay (eFigure 4 in Supplement 1).

Grouping all Black, Hispanic, and other races and ethnicities into a single group led to similar findings (eFigure 5 in Supplement 1), where Black, Hispanic, and other races and ethnicities were associated with a lower likelihood of a greater amount of EHR engagement compared with White at both VUMC (AOR, 0.86; 95% CI, 0.83-0.88; *P* < .001) and NW Medicine (AOR, 0.90; 95% CI, 0.88-0.92; *P* < .001). Across racial and ethnic subpopulations, both the outcome variable distributions (eFigure 6 in Supplement 1) and the unadjusted results (eTable 1 in Supplement 1) provided the same messages as the adjusted results.

**Figure 2.**
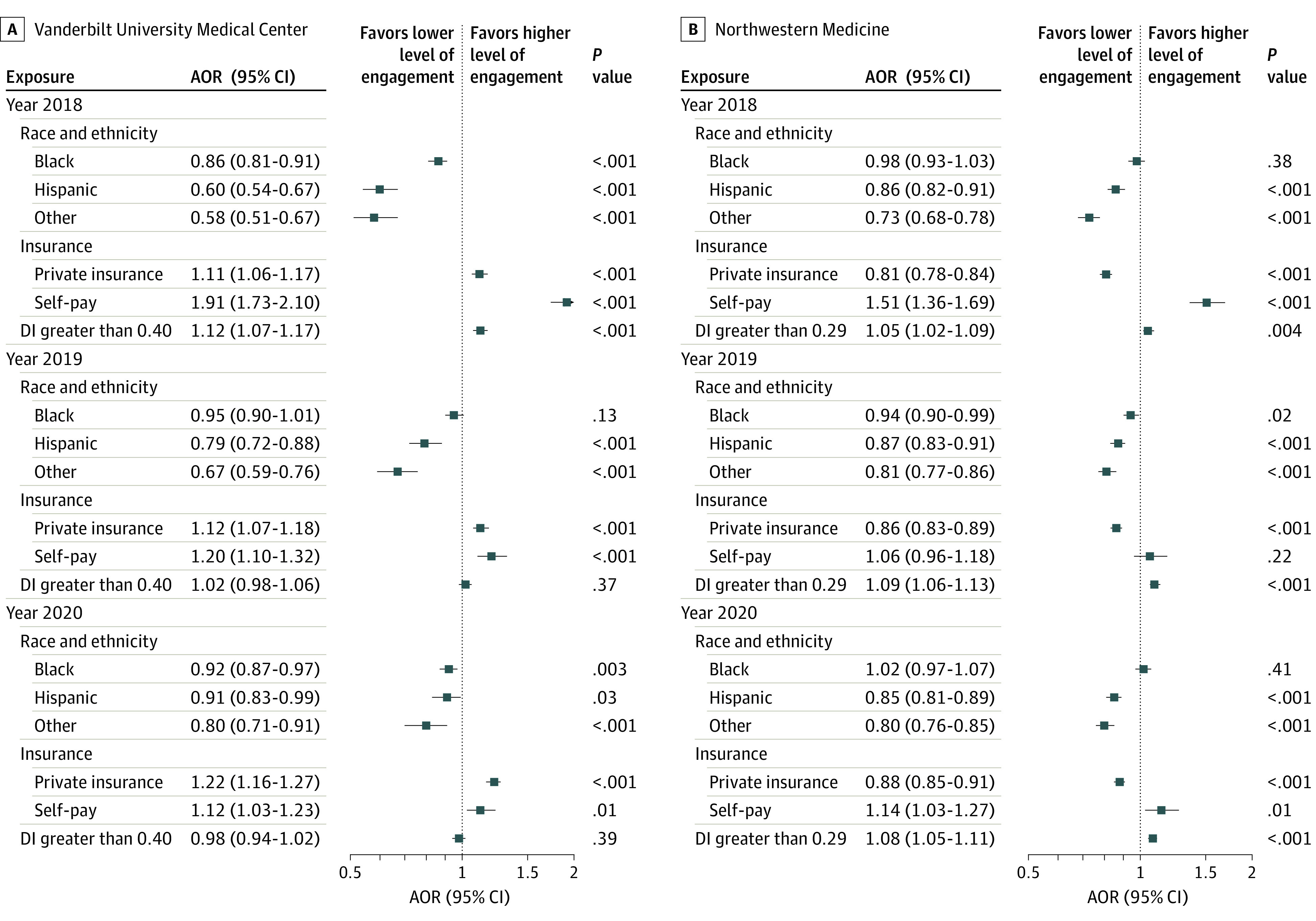
Adjusted Odds Ratios (AORs) of Patients’ Demographic and Socioeconomic Factors for Electronic Health Record Engagement in the 2018, 2019, and 2020 Cohorts White race, public insurance, and deprivation index (DI) less than 0.40 for Vanderbilt University Medical Center and less than 0.29 for Northwestern Medicine are the reference groups for race and ethnicity, insurance type, and DI, respectively. Comorbidity categories were defined per Charlson Comorbidity Index; age and sex were adjusted.

Figure 2 summarizes the AORs of race and ethnicity for EHR engagement of each single-year analysis (more details provided in eFigure 7 in Supplement 1). Significant differences were consistently observed in the 3 single-year analyses among patient subpopulations in race and ethnicity. An exception was that EHR engagement did not appear to differ between Black and White patients at (1) VUMC in 2019, (2) NW Medicine in 2018, and (3) NW Medicine in 2020.

Table 2 presents AORs in EHR engagement over time. Although significant differences in the likelihood of receiving more EHR engagement between White race and Black, Hispanic, and other races and ethnicities remained in each of the consecutive 3 years, this difference declined from 2018 (AORs, 0.77 [95% CI, 0.73-0.82; *P* < .001] for VUMC and 0.87 [95% CI, 0.84-0.90; *P* < .001] for NW Medicine) to 2020 (AORs, 0.90 [95% CI, 0.86-0.95; *P* < .001] for VUMC and 0.90 [95% CI, 0.87-0.93; *P* < .001] for NW Medicine). Permutation tests between 2018 and 2020 demonstrated that sufficient statistical evidence was present to conclude the difference in decline at VUMC (AOR difference, 0.13; *P* = .001), whereas the observed decline lacked sufficient statistical evidence for NW Medicine (AOR difference, 0.03; *P* = .09).

**Table 2.** Adjusted Odds Ratios (AORs) of Race and Ethnicity, Insurance, and Deprivation Index (DI) for Electronic Health Record Engagement in Single-Year Analyses

Factor	Vanderbilt University Medical Center								Northwestern Medicine							
	2018		2019			2020			2018			2019			2020	
	AOR (95% CI)	*P* value	AOR (95% CI)	*P* value	AOR (95% CI)		*P* value	AOR (95% CI)		*P* value	AOR (95% CI)		*P* value	AOR (95% CI)		*P* value
Black, Hispanic, and other races and ethnicities (vs White)	0.77 (0.73-0.82)	<.001	0.88 (0.84-0.93)	<.001	0.90 (0.86-0.95)		<.001	0.87 (0.84-0.90)		<.001	0.88 (0.85-0.91)		<.001	0.90 (0.87-0.93)		<.001
Private insurance (vs public insurance)	1.12 (1.07-1.18)	<.001	1.13 (1.08-1.18)	<.001	1.22 (1.16-1.27)		<.001	0.80 (0.77-0.83)		<.001	0.85 (0.82-0.89)		<.001	0.87 (0.84-0.90)		<.001
Self-pay (vs public insurance)	1.89 (1.72-2.09)	<.001	1.20 (1.10-1.31)	<.001	1.12 (1.03-1.23)		.01	1.49 (1.34-1.66)		<.001	1.05 (0.95-1.16)		.32	1.11 (1.00-1.23)		.04
DI ≥*t* (vs DI <*t*)	1.12 (1.07-1.17)	<.001	1.02 (0.98-1.07)	.28	0.98 (0.94-1.02)		.44	1.07 (1.03-1.10)		<.001	1.10 (1.07-1.13)		<.001	1.09 (1.06-1.12)		<.001

At both VUMC and NW Medicine, Black, Hispanic, or other race and ethnicity patients were consistently associated with a lower likelihood of receiving more EHR engagement for modify actions (AORs, 0.81 [95% CI, 0.78-0.84; *P* < .001] for VUMC and 0.77 [95% CI, 0.75-0.79; *P* < .001] for NW Medicine), view actions (AORs, 0.88 [95% CI, 0.85-0.91; *P* < .001] for VUMC and 0.91 [95% CI, 0.89-0.93; *P* < .001] for NW Medicine), and system actions (AORs, 0.92 [95% CI, 0.89-0.95; *P* < .001] for VUMC and 0.84 [95% CI, 0.82-0.86; *P* < .001] for NW Medicine) compared with White patients from 2018 to 2020 (Table 3). Examples of EHR actions with AORs significantly below 1 for Black, Hispanic, and other race and ethnicity patients (eTable 2 in Supplement 1) include barcode scanned, flowsheet viewed, and medication administration record accessed.

**Table 3.** Adjusted Odds Ratios (AORs) of Black, Hispanic, and Other Races and Ethnicities for Electronic Health Record (EHR) Engagement Categories

EHR engagement category	Vanderbilt University Medical Center								Northwestern Medicine							
	2018-2020		2018		2019		2020		2018-2020		2018		2019		2020	
	AOR (95% CI)	*P* value	AOR (95% CI)	*P* value	AOR (95% CI)	*P* value	AOR (95% CI)	*P* value	AOR (95% CI)	*P* value	AOR (95% CI)	*P* value	AOR (95% CI)	*P* value	AOR (95% CI)	*P* value
Modify actions	0.81 (0.78-0.84)	<.001	0.81 (0.77-0.86)	<.001	0.78 (0.74-0.82)	<.001	0.83 (0.79-0.88)	<.001	0.77 (0.75-0.79)	<.001	0.76 (0.73-0.79)	<.001	0.75 (0.72-0.77)	<.001	0.73 (0.71-0.75)	<.001
View actions	0.88 (0.85-0.91)	<.001	0.79 (0.75-0.84)	<.001	0.88 (0.84-0.93)	<.001	0.94 (0.89-0.98)	.01	0.91 (0.89-0.93)	<.001	0.87 (0.84-0.90)	<.001	0.89 (0.86-0.92)	<.001	0.92 (0.89-0.95)	<.001
Export actions	0.95 (0.92-0.98)	.002	0.88 (0.84-0.93)	<.001	1.00 (0.95-1.05)	.98	0.96 (0.91-1.01)	.10	1.47 (1.44-1.50)	<.001	1.58 (1.52-1.63)	<.001	1.33 (1.29-1.38)	<.001	1.41 (1.36-1.45)	<.001
System actions	0.92 (0.89-0.95)	<.001	0.82 (0.78-0.87)	<.001	0.92 (0.88-0.97)	.002	0.90 (0.85-0.94)	<.001	0.84 (0.82-0.86)	<.001	0.74 (0.72-0.77)	<.001	0.88 (0.85-0.90)	<.001	0.75 (0.73-0.78)	<.001

## Discussion

The findings of this retrospective cross-sectional study suggest that there may be differences in the level of health professionals’ EHR engagement for patients of different races and ethnicities in the inpatient care setting. We observed that minoritized racial and ethnic populations were more likely to receive a lower amount of EHR engagement during their inpatient stay compared with White patients. Consistent results were observed across the 2 institutions for the view, modify, and system categories of user-EHR interactions.

This study has implications for care delivery and clinical research. Enhanced EHR engagement in actions reflective of the thoroughness and meticulousness of care,^13,14,15,16^ such as reviewing charts, updating clinical notes, and maintaining active medications, aligns with the prospect of improving care delivery and health outcomes. Health professionals’ efforts devoted to patients’ EHRs could shape the quality of EHR data in terms of their accuracy and completeness. The quality of the accumulated EHR data, as one of the most critical information sources to inform clinical decision making, can then influence care on many levels, including the accuracy and timeliness of disease diagnosis, medication prescription, and treatment execution. On the other hand, EHR data (including EHR access log data^17^) have been increasingly used to support secondary analysis in health care (eg, clinical research, public health surveillance, or quality assurance). Racial and ethnic differences in EHR engagement, when neglected, could be transmitted into all downstream investigations such that biased results could be derived. Patient subpopulations with less EHR engagement could contribute fewer data points and less evidence reflecting health professionals’ behaviors. We provided an effective evaluation method and derived measures for medical institutions to monitor how EHR engagement is distributed across patient subpopulations.

There are various potential causes for the differences in EHR engagement, which can function in conjunction with one another, potentially compounding the outcome. First, patients in minoritized racial and ethnic populations have been observed to have less access to health care services than White patients.^9,29,30^ This disparity may lead to differences in the integrity, complexity, and precision of health information recorded in patients’ EHRs. When interacting with EHRs with more details, health professionals should devote more effort to performing more reviews and modifications to enable reliable decision making.

Second, language and communication barriers may be more commonly experienced by Hispanic patients and other minoritized racial and ethnic groups,^31,32^ which might partially explain the observed lower levels of EHR engagement. Normal care requests that were withheld from patients due to communication difficulties could have triggered less engagement from health professionals.

Third, EHRs are increasingly used in clinical research to facilitate cohort identification, streamline data collection, and act as part of the intervention.^33,34,35,36^ In addition to regular EHR use, study cohorts need to be routinely monitored to ensure medication or procedure adherence. However, minoritized racial and ethnic subpopulations are generally underrepresented in clinical research,^37,38,39^ which can contribute to differences in EHR engagement.

Fourth, a potential reason that cannot be ruled out is racial discrimination in health care, which has been repeatedly observed in the US medical system.^40,41,42,43^ Racial discrimination against minoritized populations may occur unintentionally or subconsciously and can manifest in various forms,^44,45,46,47^ such as implicit bias and stereotyping, which can lead to unfair allocation of health care resources.^48^ Numerous studies have provided evidence of implicit racial bias among health professionals. For example, an implicit association test conducted in the Denver, Colorado, metropolitan area revealed that primary care providers harbored unconscious biases against Latino and African American patients.^49^ Additionally, studies have shown that patients from minoritized racial and ethnic groups were less likely to be placed on liver transplant waitlists compared with White patients.^50^ An important reason was that the results of the psychosocial evaluations, a standard transplant assessment that is subject to evaluator bias, unequally influenced racial and ethnic groups’ access to these waitlists. In the context of EHR use, health professionals may have underprioritized the health needs of minoritized racial and ethnic subpopulations through less EHR engagement. While there is no evidence of overt discrimination, our study suggests that minoritized subpopulations may be at a disadvantage in terms of how health professionals distribute their care attention.

The observed decrease in the difference of health professionals’ EHR engagement with White vs Black, Hispanic, and other racial and ethnic minority patients from 2018 to 2020 (Table 2) may be due to changes in the patient population or shifts in health care utilization patterns, among other potential factors. In particular, there was a surge of COVID-19 hospitalizations at the 2 institutions in 2020, which caused a major care resource shift in which inpatient care services for other diseases, when possible, were delayed.^51,52^ Treatment of COVID-19 symptoms followed institutional standards and protocols, which may have allowed a smaller variance in EHR use patterns than for other conditions, such as chronic diseases.

In addition to the observed racial and ethnic differences in EHR engagement, we found that self-pay was associated with patients being more likely to receive more EHR engagement than those with public insurance over the 3-year study period (Figure 1). Health professionals may perceive self-pay patients as being more at risk for adverse outcomes due to their lack of financial resources and, thus, may be more vigilant in engaging with their EHR information.^53,54^ We also observed that higher DI scores were associated with patients being more likely to receive higher levels of EHR engagement (Figure 1), which may be due to patients’ increased care needs arising from challenging social circumstances. This observation suggests that in order to address social disparities, health professionals may need to dedicate more attention to patients with a worse DI, which may translate into more EHR engagement.^55,56^

### Limitations

This study has several limitations. First, we did not formally assess the association of medical history length with the EHR engagement differences among patient subpopulations, which is worth investigating in the future. Second, we did not focus on a specific medical setting or specific types of patients regarding the primary reason for hospitalization, which may lead to increased complexity when interpreting the results of the statistical analysis. Third, this study was conducted at 2 academic medical institutions only. The generalizability of the findings needs to be confirmed in other institutions with a wider range of institutional characteristics in terms of, for example, institutional type (eg, primary, secondary, or tertiary), patient subpopulation proportions, and location (eg, urban or rural). Fourth, we applied the category of other races and ethnicities to encompass all patients whose racial and ethnic information did not fall under Black, Hispanic, or White due to small group sizes. This led to limited information for each particular group. Fifth, the EHR engagement metric shares limitations with other EHR access log–based metrics.^23^ The EHR activities do not fully represent the comprehensive care processes a patient undergoes with health professionals in the physical world. It should be noted that EHR access log data may vary in their precision, sometimes being too coarse or too fine grained, in capturing clinically meaningful activities. Finally, this research analyzed all user-EHR interactions without distinguishing among direct, indirect, and administrative tasks related to patient care in the EHR. Additionally, the study did not consider the expertise, roles, or number of health professionals involved in the delivery of care, all of which might influence the findings.

## Conclusions

In this cross-sectional study of inpatient EHR engagement, our results consistently showed that White patients were more likely to receive more EHR engagement from health professionals than minoritized racial and ethnic populations. This study provides evidence of differences in how health professionals distribute their efforts to the EHRs of patients with different races and ethnicities. Our methodology has the potential to be widely applied to measure biases in EHR usage during care. Systematic efforts should be made to identify the underlying causes of these differences and their effects on care delivery, health outcomes, and downstream data analysis. Based on these findings, appropriate strategies should be implemented to mitigate any potential negative consequences to any patient subpopulation.

## Notes

Supplement 1. **eFigure 1.** Flow Diagrams of the Study Cohorts **eFigure 2.** Conceptual Framework of Factors Associated With EHR Engagement **eFigure 3.** Adjusted Odds Ratios of Patients’ Demographics, Socioeconomic Factors, and Comorbidities for EHR Engagement, 2018-2020 **eFigure 4.** Sensitivity Analysis Results Examining the Effect of Length of Stay on EHR Engagement **eFigure 5.** Adjusted Odds Ratios of Patients’ Demographics (All Non-White Races and Ethnicities Grouped), Socioeconomic Factors, and Comorbidities for EHR Engagement, 2018-2020 **eFigure 6.** Distributions of User-EHR Interaction Measures Between 2018 and 2020 **eTable 1.** Unadjusted Odds Ratios of Demographic and Socioeconomic Factors Associated With EHR Engagement **eFigure 7.** Adjusted Odds Ratios of Patients’ Demographic (Race and Ethnicity, Age, and Sex) and Socioeconomic Factors for EHR Engagement in 2018, 2019, and 2020 Cohorts **eTable 2.** Examples of the Most Commonly Logged User-EHR Interaction Types With Adjusted Odds Ratios Significantly Below 1 for Non-White Patients
eReferences

Supplement 2.
Data Sharing Statement
