# Supplementary material for: Differences in Health Professionals’ Engagement With Electronic Health Records Based on Inpatient Race and Ethnicity
Source: JAMA Netw Open. 2023 Oct 9;6(10):e2336383. doi: 10.1001/jamanetworkopen.2023.36383 (PMC10562942; doi:10.1001/jamanetworkopen.2023.36383)
Supplement: Supplement 1. — eFigure 1. Flow Diagrams of the Study Cohorts eFigure 2. Conceptual Framework of Factors Associated With EHR Engagement eFigure 3. Adjusted Odds Ratios of Patients’ Demographics, Socioeconomic Factors, and Comorbidities for EHR Engagement, 2018-2020 eFigure 4. Sensitivity Analysis Results Examining the Effect of Length of Stay on EHR Engagement eFigure 5. Adjusted Odds Ratios of Patients’ Demographics (All Non-White Races and Ethnicities Grouped), Socioeconomic Factors, and Comorbidities for EHR Engagement, 2018-2020 eFigure 6. Distributions of User-EHR Interaction Measures Between 2018 and 2020 eTable 1. Unadjusted Odds Ratios of Demographic and Socioeconomic Factors Associated With EHR Engagement eFigure 7. Adjusted Odds Ratios of Patients’ Demographic (Race and Ethnicity, Age, and Sex) and Socioeconomic Factors for EHR Engagement in 2018, 2019, and 2020 Cohorts eTable 2. Examples of the Most Commonly Logged User-EHR Interaction Types With Adjusted Odds Ratios Significantly Below 1 for Non-White Patients eReferences [file jamanetwopen-e2336383-s001.pdf]

Supplemental Online Content

Yan C, Zhang X, Yang Y, et al. Professionals' engagement with electronic health records based on inpatient race and ethnicity. *JAMA Netw Open*. 2023;6(10):e2336383. doi:10.1001/jamanetworkopen.2023.36383

eFigure 1. Flow Diagrams of the Study Cohorts

eFigure 2. Conceptual Framework of Factors Associated With EHR Engagement

eFigure 3. Adjusted Odds Ratios of Patients' Demographics, Socioeconomic Factors, and Comorbidities for EHR Engagement, 2018-2020

eFigure 4. Sensitivity Analysis Results Examining the Effect of Length of Stay on EHR Engagement

eFigure 5. Adjusted Odds Ratios of Patients' Demographics (All Non-White Races and Ethnicities Grouped), Socioeconomic Factors, and Comorbidities for EHR Engagement, 2018-2020

eFigure 6. Distributions of User-EHR Interaction Measures Between 2018 and 2020

eTable 1. Unadjusted Odds Ratios of Demographic and Socioeconomic Factors Associated With EHR Engagement

eFigure 7. Adjusted Odds Ratios of Patients' Demographic (Race and Ethnicity, Age, and Sex) and Socioeconomic Factors for EHR Engagement in 2018, 2019, and 2020 Cohorts

eTable 2. Examples of the Most Commonly Logged User-EHR Interaction Types With Adjusted Odds Ratios Significantly Below 1 for Non-White Patients

eReferences

This supplemental material has been provided by the authors to give readers additional information about their work.

© 2023 Yan C et al. *JAMA Network Open.*

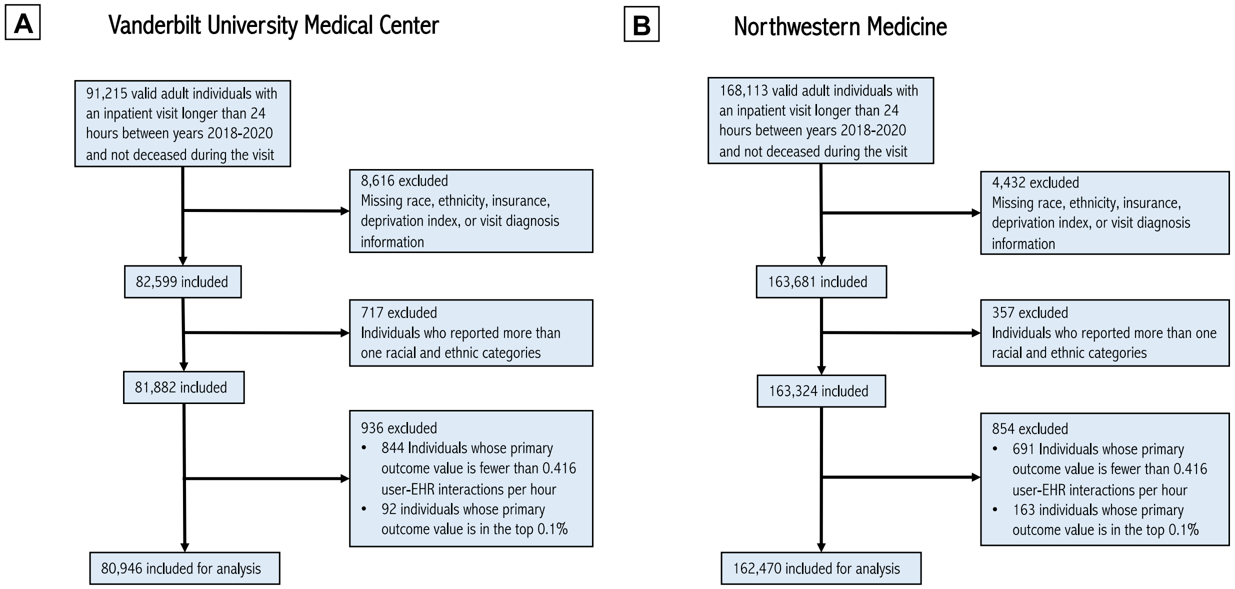

eFigure 1. Flow Diagrams of the Study Cohorts

For this study, we removed inpatient stays if it 1) was associated with more than one reported racial or ethnic category or 2) experienced less than 0.46 user-EHR interactions per hour (which is equivalent to less than 10 user-EHR interactions per day), as well as those with a primary outcome value in the top 0.1%.

Only 0.8% of patients at Vanderbilt University Medical Center and 0.2% of patients at Northwestern Medicine with an inpatient stay longer than 24 hours had more than one reported racial or ethnic categories. Given the small percentage of these cases, we chose to exclude these patients to maintain clean cohorts. This also simplified the modeling related to the race/ethnicity variable. Additionally, we excluded the inpatient stays that corresponded to the two extremes of the distribution of the dependent variable (i.e., EHR engagement). This is because we believed these outlier cases may not represent typical inpatient stays in terms of inpatient care or EHR usage.

© 2023 Yan C et al. *JAMA Network Open.*

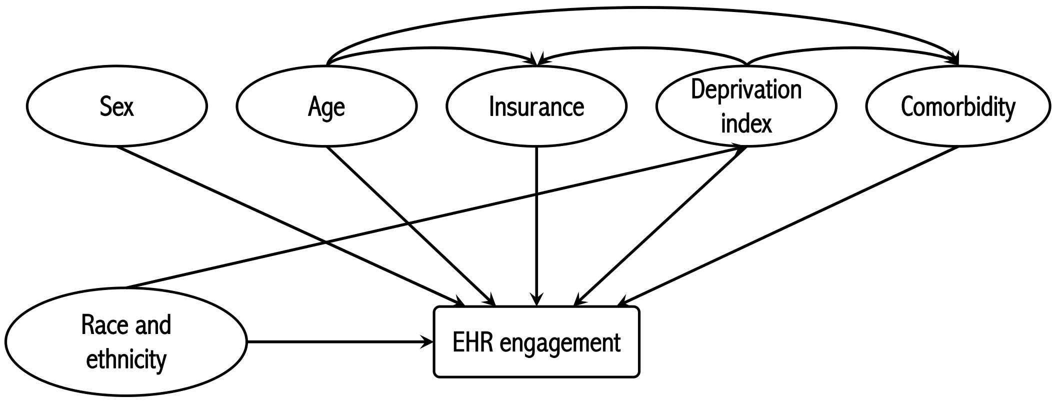

eFigure 2. Conceptual Framework of Factors Associated With EHR Engagement

The primary exposure variable and the dependent variable are race and ethnicity and EHR engagement, respectively. We incorporated sex, age, insurance types, deprivation index, and comorbidity index as covariates, which are postulated to either mediate or moderate the association between race and ethnicity and EHR engagement. Previous literature[1-5] was reviewed to identify the associations among non-dependent variables.

© 2023 Yan C et al. *JAMA Network Open.*

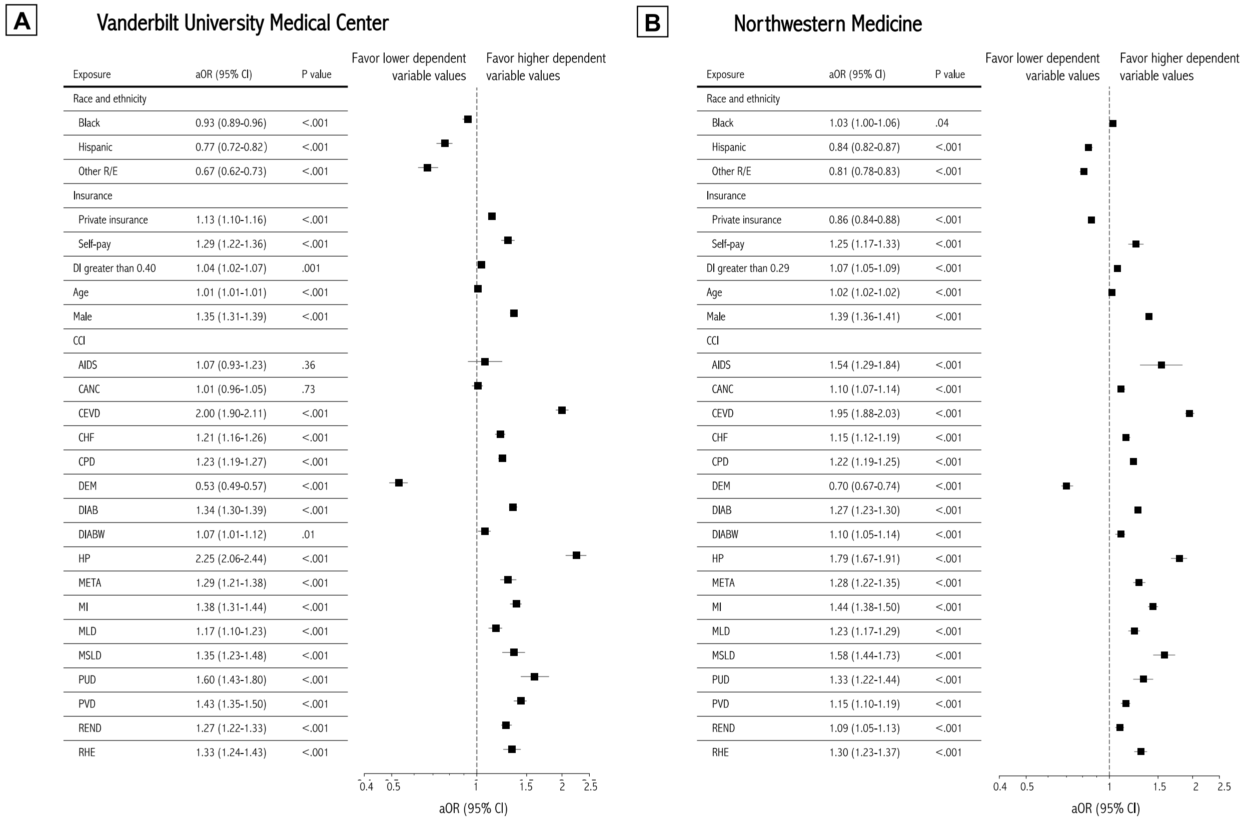

A Vanderbilt University Medical Center

Exposure	aOR (95% CI)	P value
Race and ethnicity		
Black	0.93 (0.89-0.96)	<.001
Hispanic	0.77 (0.72-0.82)	<.001
Other R/E	0.67 (0.62-0.73)	<.001
Insurance		
Private insurance	1.13 (1.10-1.16)	<.001
Self-pay	1.29 (1.22-1.36)	<.001
DI greater than 0.40	1.04 (1.02-1.07)	.001
Age	1.01 (1.01-1.01)	<.001
Male	1.35 (1.31-1.39)	<.001
CCI		
AIDS	1.07 (0.93-1.23)	.36
CANC	1.01 (0.96-1.05)	.73
CEVD	2.00 (1.90-2.11)	<.001
CHF	1.21 (1.16-1.26)	<.001
CPD	1.23 (1.19-1.27)	<.001
DEM	0.53 (0.49-0.57)	<.001
DIAB	1.34 (1.30-1.39)	<.001
DIABW	1.07 (1.01-1.12)	.01
HP	2.25 (2.06-2.44)	<.001
META	1.29 (1.21-1.38)	<.001
MI	1.38 (1.31-1.44)	<.001
MLD	1.17 (1.10-1.23)	<.001
MSLD	1.35 (1.23-1.48)	<.001
PUD	1.60 (1.43-1.80)	<.001
PVD	1.43 (1.35-1.50)	<.001
REND	1.27 (1.22-1.33)	<.001
RHE	1.33 (1.24-1.43)	<.001

B Northwestern Medicine

Exposure	aOR (95% CI)	P value
Race and ethnicity		
Black	1.03 (1.00-1.06)	.04
Hispanic	0.84 (0.82-0.87)	<.001
Other R/E	0.81 (0.78-0.83)	<.001
Insurance		
Private insurance	0.86 (0.84-0.88)	<.001
Self-pay	1.25 (1.17-1.33)	<.001
DI greater than 0.29	1.07 (1.05-1.09)	<.001
Age	1.02 (1.02-1.02)	<.001
Male	1.39 (1.36-1.41)	<.001
CCI		
AIDS	1.54 (1.29-1.84)	<.001
CANC	1.10 (1.07-1.14)	<.001
CEVD	1.95 (1.88-2.03)	<.001
CHF	1.15 (1.12-1.19)	<.001
CPD	1.22 (1.19-1.25)	<.001
DEM	0.70 (0.67-0.74)	<.001
DIAB	1.27 (1.23-1.30)	<.001
DIABW	1.10 (1.05-1.14)	<.001
HP	1.79 (1.67-1.91)	<.001
META	1.28 (1.22-1.35)	<.001
MI	1.44 (1.38-1.50)	<.001
MLD	1.23 (1.17-1.29)	<.001
MSLD	1.58 (1.44-1.73)	<.001
PUD	1.33 (1.22-1.44)	<.001
PVD	1.15 (1.10-1.19)	<.001
REND	1.09 (1.05-1.13)	<.001
RHE	1.30 (1.23-1.37)	<.001

eFigure 3. Adjusted Odds Ratios of Patients' Demographics, Socioeconomic Factors, and Comorbidities for EHR Engagement, 2018-2020

White, public insurance, DI < t (t = 0.40 for VUMC, t = 0.29 for NW Medicine), and female are the reference groups for race and ethnicity, insurance type, DI, and sex, respectively. The last section corresponds to 17 adjusted comorbidity categories defined under CCI. Other R/E = other Races/Ethnicities; DI = Deprivation index; AIDS = AIDS/HIV; CANC = cancer (any malignancy); CEVD = Cerebrovascular disease; CHF = Congestive heart failure; CPD = Chronic pulmonary disease; DEM = Dementia; DIAB = Diabetes without complications; DIABW = Diabetes with complications; HP = Hemiplegia or paraplegia; META = Metastatic solid tumors; MI = Myocardial infarction; MLD = Mild liver disease; MSLD = Moderate or severe liver disease; PUD = Peptic ulcer disease; PVD = Peripheral vascular disease; REND = Renal disease; RHE = Rheumatoid disease.

We found that female patients were associated with a higher likelihood of receiving less EHR engagement compared to male patients. However, in this study, sex was relied upon as a covariate to mitigate biases when estimating the association between the patients' races and ethnicities and the level of EHR engagement received. In our study design, the odds ratio for sex indicated an association with EHR engagement. We believe that this is due, in part, to our lack of differentiation between reasons for inpatient stays. For instance, patients may present with care needs that are sex-specific. Therefore, a future study is needed to investigate the potential differences in EHR engagement between male and female patients, accounting for these sex-specific care requests and processes.

© 2023 Yan C et al. *JAMA Network Open.*

A Vanderbilt University Medical Center

Exposure	aOR (99.5% CI)	P value
Race and ethnicity		
Black	0.92 (0.89-0.96)	<.001
Hispanic	0.77 (0.73-0.82)	<.001
Other R/E	0.68 (0.62-0.73)	<.001
Insurance		
Private insurance	1.14 (1.10-1.17)	<.001
Self-pay	1.28 (1.21-1.36)	<.001
DI greater than 0.40	1.04 (1.01-1.07)	.003
Age	1.01 (1.01-1.01)	<.001
Male	1.34 (1.30-1.37)	<.001
Length of stay (day)	1.01 (1.01-1.02)	<.001
CCI		
AIDS	1.05 (0.91-1.21)	.50
CANC	0.99 (0.95-1.04)	.69
CEVD	1.96 (1.86-2.06)	<.001
CHF	1.19 (1.14-1.24)	<.001
CPD	1.22 (1.18-1.26)	<.001
DEM	0.51 (0.47-0.55)	<.001
DIAB	1.33 (1.28-1.38)	<.001
DIABW	1.07 (1.02-1.12)	.009
HP	2.17 (1.99-2.36)	<.001
META	1.29 (1.21-1.37)	<.001
MI	1.36 (1.30-1.43)	<.001
MLD	1.16 (1.09-1.22)	<.001
MSLD	1.32 (1.20-1.45)	<.001
PUD	1.56 (1.39-1.75)	<.001
PVD	1.40 (1.33-1.48)	<.001
REND	1.25 (1.20-1.31)	<.001
RHE	1.32 (1.23-1.42)	<.001

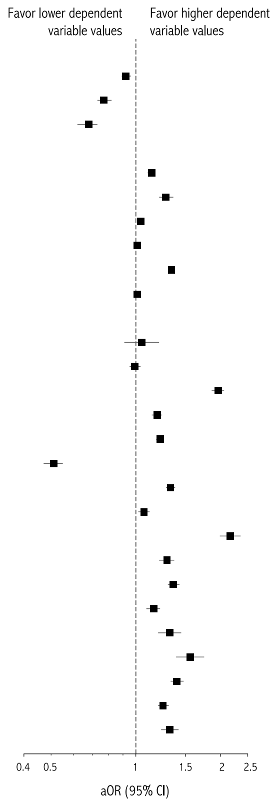

B Northwestern Medicine

Exposure	aOR (99.5% CI)	P value
Race and ethnicity		
Black	1.07 (1.04-1.10)	<.001
Hispanic	0.85 (0.82-0.87)	<.001
Other R/E	0.81 (0.78-0.84)	<.001
Insurance		
Private insurance	0.85 (0.83-0.87)	<.001
Self-pay	1.25 (1.17-1.33)	<.001
DI greater than 0.29	1.08 (1.06-1.10)	<.001
Age	1.02 (1.02-1.02)	<.001
Male	1.43 (1.40-1.46)	<.001
Length of stay (day)	0.95 (0.95-0.95)	<.001
CCI		
AIDS	1.71 (1.43-2.05)	<.001
CANC	1.21 (1.17-1.25)	<.001
CEVD	2.08 (1.99-2.16)	<.001
CHF	1.25 (1.21-1.29)	<.001
CPD	1.25 (1.22-1.28)	<.001
DEM	0.75 (0.72-0.79)	<.001
DIAB	1.32 (1.29-1.36)	<.001
DIABW	1.12 (1.08-1.17)	<.001
HP	2.15 (2.01-2.30)	<.001
META	1.32 (1.25-1.39)	<.001
MI	1.46 (1.40-1.52)	<.001
MLD	1.29 (1.23-1.35)	<.001
MSLD	1.79 (1.63-1.96)	<.001
PUD	1.50 (1.38-1.63)	<.001
PVD	1.21 (1.16-1.26)	<.001
REND	1.14 (1.10-1.18)	<.001
RHE	1.36 (1.28-1.43)	<.001

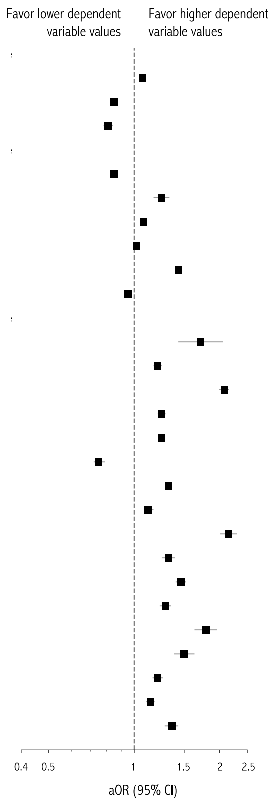

eFigure 4. Sensitivity Analysis Results Examining the Effect of Length of Stay on EHR Engagement

White, public insurance, DI < t (t = 0.40 for VUMC, t = 0.29 for NW Medicine), and female are the reference groups for race and ethnicity, insurance type, DI, and sex, respectively. The last section corresponds to 17 adjusted comorbidity categories defined under CCI. Other R/E = other Races/Ethnicities; DI = Deprivation index; AIDS = AIDS/HIV; CANC = cancer (any malignancy); CEVD = Cerebrovascular disease; CHF = Congestive heart failure; CPD = Chronic pulmonary disease; DEM = Dementia; DIAB = Diabetes without complications; DIABW = Diabetes with complications; HP = Hemiplegia or paraplegia; META = Metastatic solid tumors; MI = Myocardial infarction; MLD = Mild liver disease; MSLD = Moderate or severe liver disease; PUD = Peptic ulcer disease; PVD = Peripheral vascular disease; REND = Renal disease; RHE = Rheumatoid disease.

© 2023 Yan C et al. *JAMA Network Open.*

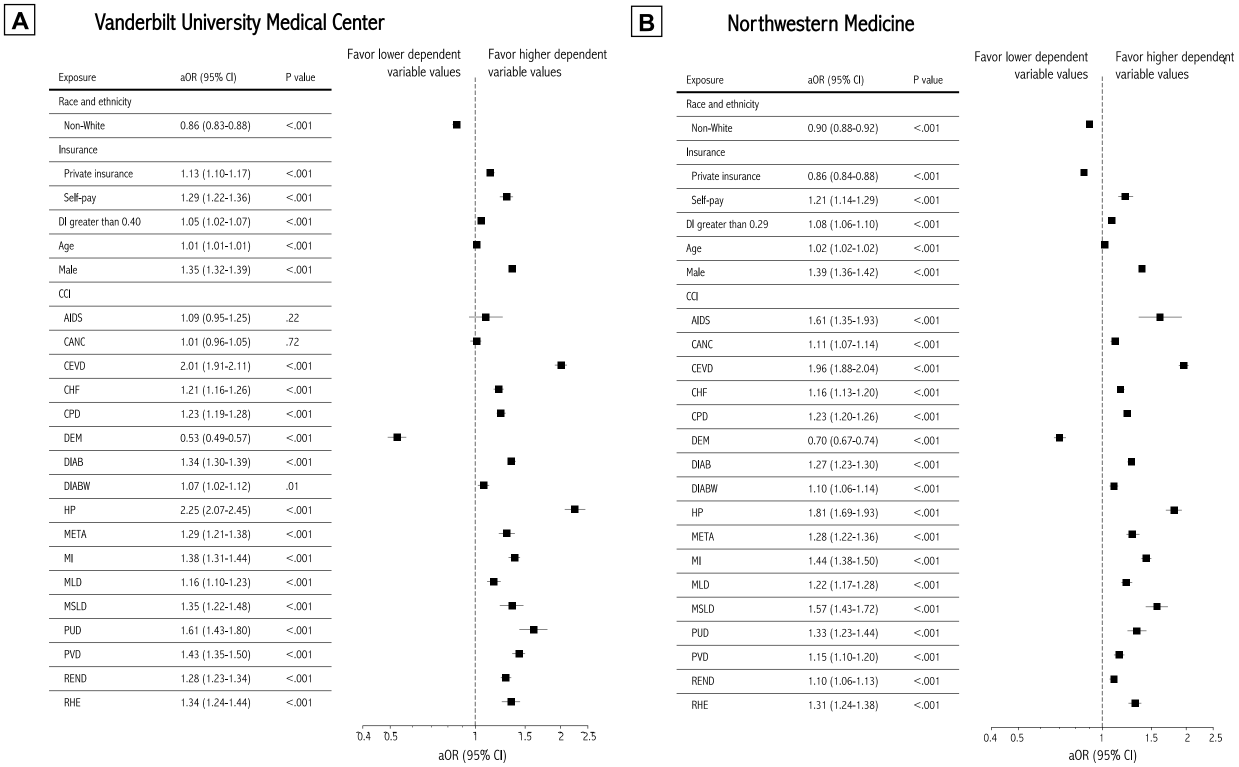

eFigure 5. Adjusted Odds Ratios of Patients' Demographics (All Non-White Races and Ethnicities Grouped), Socioeconomic Factors, and Comorbidities for EHR Engagement, 2018-2020

White, public insurance, DI < t (t = 0.40 for VUMC, t = 0.29 for NW Medicine), and female are the reference groups for race and ethnicity, insurance type, DI, and sex, respectively. The last section corresponds to 17 adjusted comorbidity categories defined under CCI. DI = Deprivation index; AIDS = AIDS/HIV; CANC = cancer (any malignancy); CEVD = Cerebrovascular disease; CHF = Congestive heart failure; CPD = Chronic pulmonary disease; DEM = Dementia; DIAB = Diabetes without complications; DIABW = Diabetes with complications; HP = Hemiplegia or paraplegia; META = Metastatic solid tumors; MI = Myocardial infarction; MLD = Mild liver disease; MSLD = Moderate or severe liver disease; PUD = Peptic ulcer disease; PVD = Peripheral vascular disease; REND = Renal disease; RHE = Rheumatoid disease.

© 2023 Yan C et al. *JAMA Network Open.*

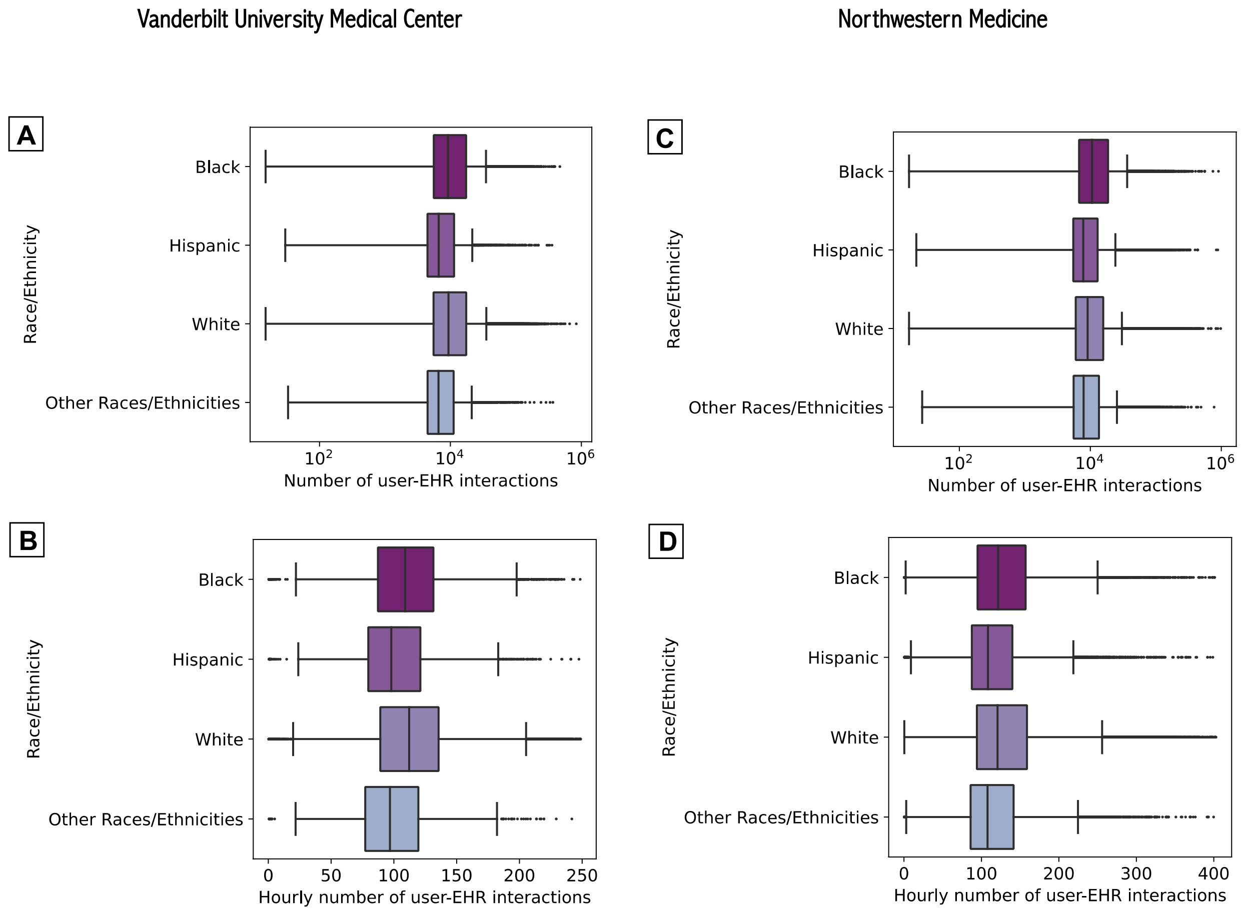

eFigure 6. Distributions of User-EHR Interaction Measures Between 2018 and 2020
A,C, the total number of use-EHR interactions. B,D, the hourly number of use-EHR interactions across racial and ethnic subpopulations.

© 2023 Yan C et al. *JAMA Network Open.*

eTable 1. Unadjusted Odds Ratios of Demographic and Socioeconomic Factors Associated With EHR Engagement

	VUMC								NW Medicine							
	2018-2020		2018		2019		2020		2018-2020		2018		2019		2020	
	OR (95%CI)	p-value	OR (95%CI)	p-value	OR (95%CI)	p-value	OR (95%CI)	p-value	OR (95%CI)	p-value	OR (95%CI)	p-value	OR (95%CI)	p-value	OR (95%CI)	p-value
**White**	1 [reference]															
**Black**	0.86 (0.83-0.89)	<.001	0.82 (0.77-0.87)	<.001	0.90 (0.85-0.95)	<.001	0.87 (0.82-0.91)	<.001	1.01 (0.98-1.04)	.57	0.95 (0.91-0.99)	.02	0.96 (0.92-1.00)	.08	1.03 (0.98-1.07)	.22
**Hispanic**	0.53 (0.50-0.56)	<.001	0.41 (0.37-0.46)	<.001	0.55 (0.50-0.60)	<.001	0.64 (0.59-0.70)	<.001	0.63 (0.61-0.65)	<.001	0.59 (0.56-0.62)	<.001	0.63 (0.60-0.66)	<.001	0.66 (0.63-0.69)	<.001
**Other R/E**	0.49 (0.45-0.53)	<.001	0.40 (0.35-0.46)	<.001	0.49 (0.43-0.56)	<.001	0.63 (0.55-0.71)	<.001	0.62 (0.60-0.64)	<.001	0.52 (0.49-0.55)	<.001	0.62 (0.59-0.66)	<.001	0.65 (0.61-0.68)	<.001
**Female**	1 [reference]															
**Male**	1.69 (1.64-1.73)	<.001	1.95 (1.87-2.04)	<.001	1.64 (1.57-1.71)	<.001	1.47 (1.42-1.53)	<.001	2.12 (2.08-2.16)	<.001	2.77 (2.68-2.86)	<.001	2.18 (2.11-2.24)	<.001	1.92 (1.87-1.98)	<.001
**Public insurance**	1 [reference]															
**Private insurance**	0.85 (0.83-0.87)	<.001	0.81 (0.77-0.84)	<.001	0.85 (0.81-0.88)	<.001	0.95 (0.92-0.99)	.03	0.44 (0.43-0.44)	<.001	0.32 (0.31-0.33)	<.001	0.42 (0.40-0.43)	<.001	0.47 (0.45-0.48)	<.001
**Self-pay**	1.02 (0.96-1.07)	.52	1.43 (1.30-1.56)	<.001	0.94 (0.86-1.03)	.19	0.89 (0.81-0.97)	.007	0.79 (0.74-0.84)	<.001	0.77 (0.70-0.85)	<.001	0.67 (0.61-0.74)	<.001	0.76 (0.69-0.84)	<.001
**DI<t**	1 [reference]															
**DI>=t**	1.06 (1.04-1.09)	<.001	1.13 (1.09-1.18)	<.001	1.04 (1.00-1.08)	.06	1.01 (0.97-1.05)	.79	1.00 (0.99-1.02)	.59	0.98 (0.95-1.01)	.15	1.01 (0.98-1.03)	.71	1.02 (0.99-1.04)	.25

Other R/E = other Races/Ethnicities; DI = Deprivation index.

© 2023 Yan C et al. *JAMA Network Open.*

A Vanderbilt University Medical Center

Exposure	aOR (95% CI)	P value
Year 2018		
Race and ethnicity		
Black	0.86 (0.81-0.91)	<.001
Hispanic	0.60 (0.54-0.67)	<.001
Other R/E	0.58 (0.51-0.67)	<.001
Insurance		
Private insurance	1.11 (1.06-1.17)	<.001
Self-pay	1.91 (1.73-2.10)	<.001
DI greater than 0.40	1.12 (1.07-1.17)	<.001
Age	1.01 (1.01-1.01)	<.001
Male	1.53 (1.47-1.60)	<.001
Year 2019		
Race and ethnicity		
Black	0.95 (0.90-1.01)	.13
Hispanic	0.79 (0.72-0.88)	<.001
Other R/E	0.67 (0.59-0.76)	<.001
Insurance		
Private insurance	1.12 (1.07-1.18)	<.001
Self-pay	1.20 (1.10-1.32)	<.001
DI greater than 0.40	1.02 (0.98-1.06)	.37
Age	1.01 (1.01-1.01)	<.001
Male	1.33 (1.28-1.39)	<.001
Year 2020		
Race and ethnicity		
Black	0.92 (0.87-0.97)	.003
Hispanic	0.91 (0.83-0.99)	.03
Other R/E	0.80 (0.71-0.91)	<.001
Insurance		
Private insurance	1.22 (1.16-1.27)	<.001
Self-pay	1.12 (1.03-1.23)	.01
DI greater than 0.40	0.98 (0.94-1.02)	.39
Age	1.01 (1.01-1.01)	<.001
Male	1.21 (1.16-1.26)	<.001

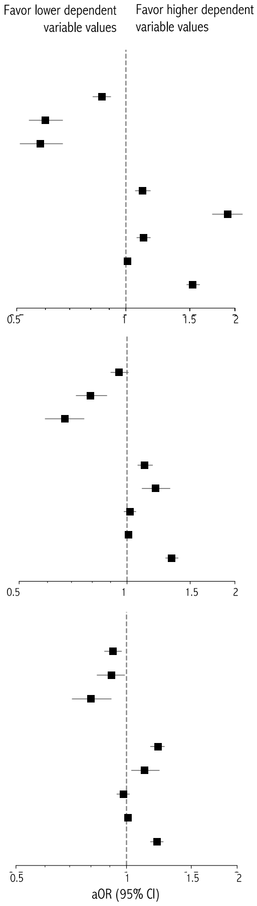

B Northwestern Medicine

Exposure	aOR (95% CI)	P value
Year 2018		
Race and ethnicity		
Black	0.98 (0.93-1.03)	.38
Hispanic	0.86 (0.82-0.91)	<.001
Other R/E	0.73 (0.68-0.78)	<.001
Insurance		
Private insurance	0.81(0.78-0.84)	<.001
Self-pay	1.51 (1.36-1.69)	<.001
DI greater than 0.29	1.05 (1.02-1.09)	.004
Age	1.03 (1.03-1.03)	<.001
Male	1.72 (1.66-1.78)	<.001
Year 2019		
Race and ethnicity		
Black	0.94 (0.90-0.99)	.02
Hispanic	0.87 (0.83-0.91)	<.001
Other R/E	0.81 (0.77-0.86)	<.001
Insurance		
Private insurance	0.86 (0.83-0.89)	<.001
Self-pay	1.06 (0.96-1.18)	.22
DI greater than 0.29	1.09 (1.06-1.13)	<.001
Age	1.02 (1.02-1.02)	<.001
Male	1.44 (1.40-1.49)	<.001
Year 2020		
Race and ethnicity		
Black	1.02 (0.97-1.07)	.41
Hispanic	0.85 (0.81-0.89)	<.001
Other R/E	0.80 (0.76-0.85)	<.001
Insurance		
Private insurance	0.88 (0.85-0.91)	<.001
Self-pay	1.14 (1.03-1.27)	.01
DI greater than 0.29	1.08 (1.05-1.11)	<.001
Age	1.01 (1.01-1.02)	<.001
Male	1.27 (1.24-1.32)	<.001

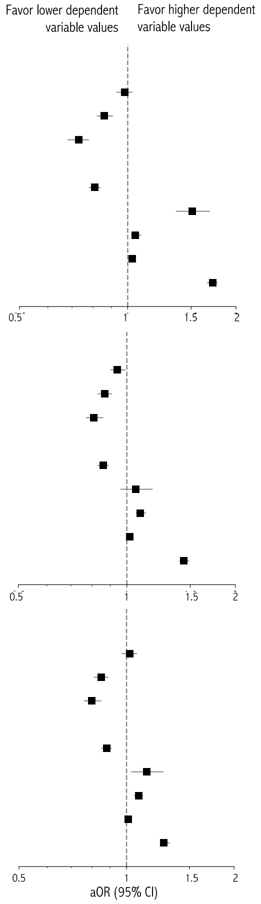

eFigure 7. Adjusted Odds Ratios of Patients' Demographic (Race and Ethnicity, Age, and Sex) and Socioeconomic Factors for EHR Engagement in 2018, 2019, and 2020 Cohorts

White, public insurance, DI < t (t = 0.40 for VUMC, t = 0.29 for NW Medicine), and female are the reference groups for race and ethnicity, insurance type, DI, and sex, respectively. Comorbidity categories defined under CCI were adjusted. Other R/E = other Races/Ethnicities; DI = Deprivation index.

© 2023 Yan C et al. *JAMA Network Open.*

eTable 2. Examples of the Most Commonly Logged User-EHR Interaction Types With Adjusted Odds Ratios Significantly Below 1 for Non-White Patients

Action Name	Description	Category	Vanderbilt University Medical Center			Northwestern Medicine		
			aOR	95% CI	*p*-value	aOR	95% CI	*p*-value
**Barcode scanned**	Scan barcode during workflows, such as patient registration or order verification.	System	0.68	0.66-0.70	<.001	0.76	0.74-0.77	<.001
**Flowsheet viewed**	View a flowsheet record, which contains the vitals or lab results of a patient.	View	0.96	0.93-0.99	.01	0.85	0.83-0.87	<.001
**MAR accessed**	Access a medication administration record (MAR), which allows monitoring and management of an inpatient medication administration.	View	0.72	0.70-0.74	<.001	0.91	0.89-0.93	<.001
**MAR administration viewed**	View a medication administration record.	View	0.71	0.69-0.73	<.001	0.78	0.76-0.79	<.001
**Report with patient data viewed**	View a document that contains patient data. Examples generating this action include viewing the patient history tab, health maintenance, Best Practice Advisory review, and patient station	View	0.91	0.88-0.94	<.001	0.85	0.83-0.87	<.001
**SmartText used**	Use a standard template of text during documentation.	View	0.95	0.92-0.98	.004	0.97	0.91-1.00	.02
**Visit Navigator template loaded**	Refer to the loading of the template used to display information in a section of the patient chart. This audit stamp is displayed when the template is loaded, such as when navigating through the patient Snapshot, History, or Episode of Care sections.	View	0.91	0.88-0.94	<.001	0.90	0.88-0.91	<.001

We provided a list of user-EHR interaction types common to both Vanderbilt University Medical Center and Northwestern Medicine that rank among the top 20 most frequently logged interaction types. The analyses, spanning the period from 2018 to 2020, utilized each user-EHR interaction type as the dependent variable by grouping all non-White racial and ethnic categories into a single group (i.e., non-White).

© 2023 Yan C et al. *JAMA Network Open.*

eReferences

1. Dhongde S, Dong X. Analyzing racial and ethnic differences in the USA through the lens of multidimensional poverty. J Econ Race Pol. 2022;5(4):252-266.
2. Cohen RA, Cha AE, Terlizzi EP, Martinez ME. Demographic variation in health insurance coverage: United States, 2019. Natl Health Stat Report. 2021;(159):1-15.
3. Brokamp C, Beck AF, Goyal NK, Ryan P, Greenberg JM, Hall ES. Material community deprivation and hospital utilization during the first year of life: an urban population-based cohort study. Ann Epidemiol. 2019;30:37-43.
4. McLean G, Gunn J, Wyke S, et al. The influence of socioeconomic deprivation on multimorbidity at different ages: a cross-sectional study. Br J Gen Pract. 2014;64(624):e440-7.
5. Piccirillo JF, Vlahiotis A, Barrett LB, Flood KL, Spitznagel EL, Steyerberg EW. The changing prevalence of comorbidity across the age spectrum. Crit Rev Oncol Hematol. 2008;67(2):124-132.

© 2023 Yan C et al. *JAMA Network Open.*
