# Supplementary material for: Differences in Health Professionals’ Engagement With Electronic Health Records Based on Inpatient Race and Ethnicity
Source: JAMA Netw Open. 2023 Oct 9;6(10):e2336383. doi: 10.1001/jamanetworkopen.2023.36383 (PMC10562942; doi:10.1001/jamanetworkopen.2023.36383)
Supplement: Supplement 2. — Data Sharing Statement [file jamanetwopen-e2336383-s002.pdf]

Data Sharing Statement

Yan. Differences in Health Professionals' Engagement With Electronic Health Records Based on Inpatient Race and Ethnicity. *JAMA Netw Open*. Published October 09, 2023. doi:10.1001/jamanetworkopen.2023.36383

Data
Data available: Yes
Data types: Data dictionary, Other (please specify)
Additional Information: We are developing a Data Sharing Plan whereby interested investigators of medical institutions can apply for access to the data through a defined proposal process. For those interested in accessing the data, please email the corresponding authors.
How to access data: you.chen@vumc.org
When available: With publication

Supporting Documents
Document types: Other (please specify)
Additional Information: We are developing a Data Sharing Plan whereby interested investigators of medical institutions can apply for access to the data through a defined proposal process. For those interested in accessing the data, please email the corresponding authors.
How to access documents: you.chen@vumc.org
When available: With publication

Additional Information
Who can access the data: Researchers whose proposed use of the data has been approved.
Types of analyses: EHR usage and clinician burden related study.
Mechanisms of data availability: With a signed data access agreement.
